# 2,3:4,5-Di-*O*-isopropyl­idenefructos-1-yl *p*-toluene­sulfonate

**DOI:** 10.1107/S1600536810044582

**Published:** 2010-11-06

**Authors:** Shiyong Huo, Yueqing Li, Chaoyan Liang, Jihong Liu, Weijie Zhao

**Affiliations:** a School of Pharmaceutical Science and Technology, Dalian University of Technology, PO Box 90, Zhongshan Road 158, Dalian 116012, People’s Republic of China

## Abstract

The title compound, C_19_H_26_O_8_S, has been synthesized from 2,3:4,5-di-*O*-isopropyl­idene-β-d-fructopyran­ose. The absolute configuration of the fused ring is confirmed by anomalous dispersion effects in the diffraction measurement. The six-membered β-fructopyran­ose ring has a twist-boat conformation with the two five-membered rings *trans* to each other. In the crystal, inter­molecular non-classical C—H⋯O hydrogen bonds link the mol­ecules into a three-dimensional network.

## Related literature

For details of the synthesis of the title compound and its analogues, see: Hirst *et al.* (1953 ▶); Reitz *et al.* (1989 ▶); Dekany *et al.* (2007 ▶). For a related structure, see: Lis & Weichsel (1987 ▶).
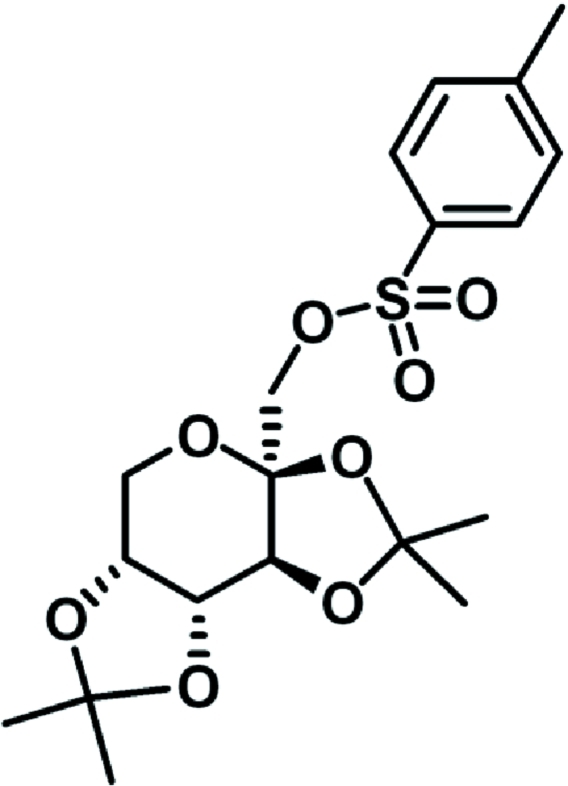


## Experimental

### 

#### Crystal data

C_19_H_26_O_8_S
*M* *_r_* = 414.47
Monoclinic, 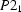

*a* = 13.870 (5) Å
*b* = 10.153 (4) Å
*c* = 15.715 (6) Å
β = 106.831 (4)°
*V* = 2118.2 (14) Å^3^
*Z* = 4
Mo *K*α radiation
μ = 0.19 mm^−1^
*T* = 273 K
0.43 × 0.36 × 0.27 mm

#### Data collection

Bruker SMART APEX CCD diffractometer
10503 measured reflections
6948 independent reflections
6040 reflections with *I* > 2σ(*I*)
*R*_int_ = 0.018

#### Refinement

*R*[*F* ^2^ > 2σ(*F* ^2^)] = 0.034
*wR*(*F* ^2^) = 0.088
*S* = 1.04
6948 reflections
506 parameters
13 restraints
H-atom parameters constrained
Δρ_max_ = 0.15 e Å^−3^
Δρ_min_ = −0.19 e Å^−3^
Absolute structure: Flack (1983 ▶), 3010 Friedel pairs
Flack parameter: −0.02 (5)

### 

Data collection: *APEX2* (Bruker, 2005 ▶); cell refinement: *SAINT-Plus* (Bruker, 2001 ▶); data reduction: *SAINT-Plus*; program(s) used to solve structure: *SHELXS97* (Sheldrick, 2008 ▶); program(s) used to refine structure: *SHELXL97* (Sheldrick, 2008 ▶); molecular graphics: *SHELXTL* (Sheldrick, 2008 ▶); software used to prepare material for publication: *SHELXTL*.

## Supplementary Material

Crystal structure: contains datablocks I, global. DOI: 10.1107/S1600536810044582/rk2243sup1.cif

Structure factors: contains datablocks I. DOI: 10.1107/S1600536810044582/rk2243Isup2.hkl

Additional supplementary materials:  crystallographic information; 3D view; checkCIF report

## Figures and Tables

**Table 1:** Hydrogen-bond geometry (Å, °)

*D*—H⋯*A*	*D*—H	H⋯*A*	*D*⋯*A*	*D*—H⋯*A*
C3—H3*A*⋯O13^i^	0.98	2.70	3.358 (3)	125
C4—H4*A*⋯C17^i^	0.98	2.83	3.657 (5)	142
C21—H21*A*⋯O3^ii^	0.98	2.58	3.456 (3)	149
C23—H23*A*⋯O16^ii^	0.98	2.62	3.466 (3)	145
C23—H23*A*⋯C33^ii^	0.98	2.89	3.738 (4)	145
C37—H37*A*⋯O8	0.93	2.61	3.341 (4)	136

Symmetry codes: (i) 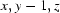; (ii) 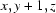.

**Fig. 1.**
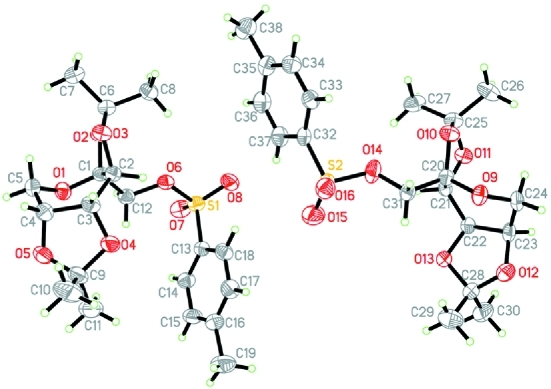
The structure of two molecules of title compound with the atom numbering scheme. Displacement ellipsoids are drawn at 30% probability level. H atoms are presented as a small spheres of arbitrary radius.

**Fig. 2.**
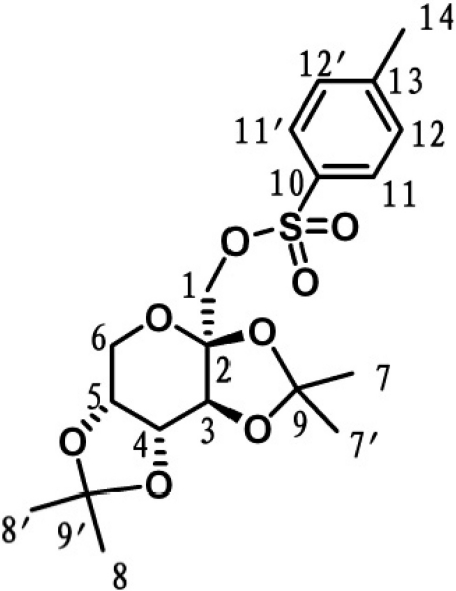
The structure of title compound, with atoms labeling corresponding to the characterization by 1H NMR and 13C NMR.

## supplementary crystallographic information

### Comment

As we know, *p*-toluenesulfonyl group has been extensively approved as a good substituent group for a long time. A series of derivatives can be synthesized by the substituent reaction of the title compound (Reitz *et al.*, 1989; Dekany *et al.*, 2007). Furthermore, in our study we found that it also can easily react with pyrrole which will be one effective approach to synthesize the corresponding novel *N*-carbohydrate-derived pyrrole compound. The molecular and crystal structure is helpful to confirm the absolute configuration of the fused ring in the derivatives of the title compound.

In the title compound, as shown at Fig. 1, the pyranose ring adopts a twist boat conformation. This conformation is the result of distortion introduced by the fusion of one six- and two five-membered rings. All bond lengths and angles of this part are normal and comparable with those reported for the related structure (Lis & Weichsel, 1987). In the crystal, weak C—H···O non-classical H bonds (Table 1) link the molecules into a three-dimensional network.

### Experimental

The title compound was prepared according to literature (Reitz *et al.*, 1989). The product (1 g) was dissolved in ethyl ether (25 ml) and hexane (25 ml). Single crystals suitable for X-ray diffraction experiment were obtained from the solution by cooling at 255 K for three days.

The molecule is characterized by NMR (Fig. 2). ^1^H NMR (CDCl_3_, 400 MHz): δ 7.79 (2*H*, d, *J* = 8.2 Hz, H-11, H-11'), 7.34 (2*H*, d, *J* = 8.2 Hz, H-12, H-12'), 4.56 (1*H*, dd, *J* = 7.9 Hz, *J* = 2.5 Hz, H-4), 4.30 (1*H*, d, *J* = 2.5 Hz, H-5), 4.20 (1*H*, d, *J* = 7.9 Hz, H-3), 4.07 (1*H*, d, *J* = 10.3 Hz, H-1a), 4.01 (1*H*, d, *J* = 10.3 Hz, H-1b), 3.86 (1*H*, d, *J* = 12.8 Hz, H-6a), 3.80(1*H*, d, *J* = 12.8 Hz, H-6b), 2.44 (3*H*, s, H-14), 1.50, 1.36, 1.31 (3*H*, 6H, 3H, CH_3_-7, CH_3_-7', CH_3_-8, CH_3_-8').

^13^C NMR(CDCl_3_, 100 MHz): δ 144.97 (C-10), 132.51(C-13), 129.84 (C-12, C-12'), 128.14 (C-11, C-11'), 109.18, 109.04 (C-9, C-9'), 100.67 (C-2), 70.59 (C-3), 69.90 (C-4, C-5), 69.04 (C-1), 61.26 (C-6), 26.44, 25.72, 25.17, 23.99 (CH_3_-7, CH_3_-7', CH_3_-8, CH_3_-8'), 21.62 (C-14).

HRMS(ES^+^): *m/z* [*M*+Na]^+^ calcd. for C_19_H_26_O_8_SNa: 437.1246; found: 437.1261.

### Refinement

All H atoms attached to C atoms were treated as riding, with C—H = 0.9700Å for methylene group, C—H = 0.9800Å for methyne group and C—H = 0.9600Å for methyl group, with *U*_iso_(H) = 1.2*U*_eq_(C) of the carrier atoms to which they are attached and *U*_iso_(H) = 1.5*U*_eq_(C) for the methyl groups. The number of Friedel pairs is 3010 which is determined by the difference between the number of unique reflections used in *SHELXL* when a 'MERG 2' and 'MERG 3' instruction were used.

### Crystal data

**Table:**

C_19_H_26_O_8_S	*F*(000) = 880
*M**_r_* = 414.47	*D*_x_ = 1.300 Mg m^−^^3^
Monoclinic, *P*2_1_	Melting point: 357 K
Hall symbol: P 2yb	Mo *K*α radiation, λ = 0.71073 Å
*a* = 13.870 (5) Å	Cell parameters from 4356 reflections
*b* = 10.153 (4) Å	θ = 2.4–23.0°
*c* = 15.715 (6) Å	µ = 0.19 mm^−^^1^
β = 106.831 (4)°	*T* = 273 K
*V* = 2118.2 (14) Å^3^	Block, colourless
*Z* = 4	0.43 × 0.36 × 0.27 mm

### Data collection

**Table:**

Bruker SMART APEX CCD diffractometer	6040 reflections with *I* > 2σ(*I*)
Radiation source: fine-focus sealed tube	*R*_int_ = 0.018
graphite	θ_max_ = 25.0°, θ_min_ = 2.3°
φ– and ω–scans	*h* = −16→9
10503 measured reflections	*k* = −12→12
6948 independent reflections	*l* = −18→18

### Refinement

**Table:**

Refinement on *F*^2^	Hydrogen site location: inferred from neighbouring sites
Least-squares matrix: full	H-atom parameters constrained
*R*[*F*^2^ > 2σ(*F*^2^)] = 0.034	*w* = 1/[σ^2^(*F*_o_^2^) + (0.0498*P*)^2^ + 0.015*P*] where *P* = (*F*_o_^2^ + 2*F*_c_^2^)/3
*wR*(*F*^2^) = 0.088	(Δ/σ)_max_ = 0.001
*S* = 1.04	Δρ_max_ = 0.15 e Å^−^^3^
6948 reflections	Δρ_min_ = −0.19 e Å^−^^3^
506 parameters	Extinction correction: *SHELXL97* (Sheldrick, 2008), Fc^*^=kFc[1+0.001xFc^2^λ^3^/sin(2θ)]^-1/4^
13 restraints	Extinction coefficient: 0.0092 (7)
Primary atom site location: structure-invariant direct methods	Absolute structure: Flack (1983), 3010 Friedel pairs
Secondary atom site location: difference Fourier map	Flack parameter: −0.02 (5)

### Special details

**Table:**

Geometry. All s.u.'s (except the s.u. in the dihedral angle between two l.s. planes) are estimated using the full covariance matrix. The cell s.u.'s are taken into account individually in the estimation of s.u.'s in distances, angles and torsion angles; correlations between s.u.'s in cell parameters are only used when they are defined by crystal symmetry. An approximate (isotropic) treatment of cell s.u.'s is used for estimating s.u.'s involving l.s. planes.
Refinement. Refinement of *F*^2^ against ALL reflections. The weighted *R*-factor *wR* and goodness of fit *S* are based on *F*^2^, conventional *R*-factors *R* are based on *F*, with *F* set to zero for negative *F*^2^. The threshold expression of *F*^2^ > σ(*F*^2^) is used only for calculating *R*-factors(gt) *etc*. and is not relevant to the choice of reflections for refinement. *R*-factors based on *F*^2^ are statistically about twice as large as those based on *F*, and *R*-factors based on ALL data will be even larger.

### Fractional atomic coordinates and isotropic or equivalent isotropic displacement parameters (Å^2^)

**Table:**

	*x*	*y*	*z*	*U*_iso_*/*U*_eq_	
S1	0.24587 (4)	0.18747 (6)	0.02021 (4)	0.05469 (18)	
O3	0.19989 (12)	−0.26856 (17)	0.18097 (10)	0.0516 (4)	
O2	0.15726 (12)	−0.22131 (18)	0.03390 (10)	0.0503 (4)	
O6	0.22579 (12)	0.04415 (17)	0.05015 (11)	0.0543 (4)	
O5	0.50852 (14)	−0.3218 (2)	0.16864 (15)	0.0765 (6)	
C1	0.25987 (17)	−0.1891 (2)	0.06751 (16)	0.0449 (5)	
O4	0.45357 (13)	−0.1488 (2)	0.23044 (13)	0.0684 (5)	
C2	0.27894 (17)	−0.1881 (3)	0.16876 (15)	0.0469 (6)	
H2A	0.2720	−0.0982	0.1890	0.056*	
C13	0.36794 (17)	0.2246 (2)	0.08676 (16)	0.0469 (6)	
C12	0.2830 (2)	−0.0621 (3)	0.02666 (18)	0.0558 (7)	
H12A	0.3545	−0.0430	0.0485	0.067*	
H12B	0.2652	−0.0710	−0.0375	0.067*	
C14	0.4482 (2)	0.2255 (3)	0.05212 (18)	0.0602 (7)	
H14A	0.4393	0.2034	−0.0071	0.072*	
C3	0.37782 (19)	−0.2460 (3)	0.22273 (17)	0.0554 (6)	
H3A	0.3737	−0.2705	0.2819	0.066*	
C8	0.0499 (2)	−0.1399 (3)	0.11720 (18)	0.0591 (7)	
H8A	0.0892	−0.0606	0.1290	0.089*	
H8B	0.0236	−0.1581	0.1662	0.089*	
H8C	−0.0049	−0.1289	0.0639	0.089*	
O8	0.17471 (14)	0.26665 (19)	0.04647 (15)	0.0734 (6)	
O7	0.24594 (14)	0.1835 (2)	−0.06986 (12)	0.0739 (5)	
C6	0.11497 (18)	−0.2526 (3)	0.10546 (15)	0.0482 (6)	
C4	0.4142 (2)	−0.3638 (3)	0.1795 (2)	0.0619 (7)	
H4A	0.4240	−0.4408	0.2186	0.074*	
C18	0.3815 (2)	0.2560 (3)	0.17503 (18)	0.0604 (7)	
H18A	0.3272	0.2549	0.1985	0.073*	
C16	0.5571 (2)	0.2930 (3)	0.1936 (2)	0.0713 (8)	
C17	0.4754 (2)	0.2886 (3)	0.2274 (2)	0.0702 (8)	
H17A	0.4847	0.3083	0.2870	0.084*	
C11	0.6036 (3)	−0.1244 (4)	0.1876 (3)	0.0982 (12)	
H11A	0.5631	−0.0963	0.1300	0.147*	
H11B	0.6621	−0.1697	0.1818	0.147*	
H11C	0.6241	−0.0491	0.2254	0.147*	
C7	0.0593 (2)	−0.3813 (3)	0.0869 (2)	0.0660 (7)	
H7B	0.1044	−0.4494	0.0799	0.099*	
H7C	0.0045	−0.3739	0.0334	0.099*	
H7D	0.0335	−0.4029	0.1356	0.099*	
C15	0.5421 (2)	0.2597 (3)	0.1066 (2)	0.0726 (8)	
H15A	0.5967	0.2601	0.0834	0.087*	
C9	0.5432 (2)	−0.2156 (3)	0.2276 (2)	0.0689 (8)	
C10	0.6013 (3)	−0.2650 (5)	0.3194 (2)	0.1077 (14)	
H10A	0.5595	−0.3235	0.3411	0.162*	
H10B	0.6208	−0.1915	0.3591	0.162*	
H10C	0.6605	−0.3111	0.3158	0.162*	
C19	0.6594 (3)	0.3360 (5)	0.2510 (3)	0.1266 (17)	
H19A	0.7068	0.3326	0.2170	0.190*	
H19B	0.6812	0.2782	0.3013	0.190*	
H19C	0.6553	0.4245	0.2712	0.190*	
S2	0.20695 (5)	0.40282 (6)	0.37302 (5)	0.05712 (18)	
O11	0.02664 (12)	0.86733 (17)	0.33953 (11)	0.0502 (4)	
O10	0.03125 (11)	0.70729 (17)	0.44201 (10)	0.0522 (4)	
O13	0.29576 (12)	0.88143 (16)	0.38639 (11)	0.0529 (4)	
C22	0.19864 (19)	0.9338 (2)	0.37952 (16)	0.0453 (6)	
H22A	0.1779	0.9944	0.3289	0.054*	
O9	0.17593 (13)	0.80561 (16)	0.53013 (10)	0.0518 (4)	
O14	0.15205 (17)	0.53361 (18)	0.38479 (14)	0.0729 (6)	
O16	0.24890 (14)	0.34602 (18)	0.45814 (13)	0.0661 (5)	
C32	0.1013 (2)	0.3142 (2)	0.31216 (17)	0.0509 (6)	
C24	0.1586 (2)	0.9447 (2)	0.52606 (17)	0.0549 (6)	
H24A	0.1823	0.9818	0.5854	0.066*	
H24B	0.0870	0.9619	0.5032	0.066*	
O12	0.31802 (15)	1.0050 (2)	0.50883 (13)	0.0710 (6)	
C28	0.3671 (2)	0.9641 (3)	0.4459 (2)	0.0602 (7)	
C31	0.1972 (2)	0.6168 (2)	0.46030 (17)	0.0509 (6)	
H31A	0.2656	0.6396	0.4617	0.061*	
H31B	0.1991	0.5717	0.5151	0.061*	
C25	−0.03423 (18)	0.7689 (3)	0.36376 (16)	0.0541 (6)	
C21	0.12717 (17)	0.8185 (2)	0.36592 (15)	0.0407 (5)	
H21A	0.1378	0.7603	0.3197	0.049*	
C20	0.13331 (17)	0.7389 (2)	0.45007 (15)	0.0428 (5)	
C34	−0.0497 (2)	0.1991 (3)	0.30565 (18)	0.0626 (7)	
H34A	−0.0964	0.1699	0.3335	0.075*	
C23	0.2126 (2)	1.0091 (2)	0.46710 (18)	0.0552 (7)	
H23A	0.1900	1.1005	0.4549	0.066*	
C35	−0.0641 (2)	0.1689 (3)	0.21767 (18)	0.0613 (7)	
C27	−0.0711 (2)	0.6678 (4)	0.29181 (19)	0.0744 (8)	
H27A	−0.0145	0.6272	0.2788	0.112*	
H27B	−0.1128	0.7098	0.2393	0.112*	
H27C	−0.1095	0.6020	0.3115	0.112*	
C36	0.0060 (2)	0.2146 (3)	0.17747 (19)	0.0702 (8)	
H36A	−0.0027	0.1955	0.1178	0.084*	
C26	−0.1181 (2)	0.8387 (4)	0.3889 (2)	0.0783 (9)	
H26A	−0.0899	0.9020	0.4348	0.117*	
H26B	−0.1571	0.7756	0.4103	0.117*	
H26C	−0.1606	0.8830	0.3378	0.117*	
C30	0.3934 (3)	1.0791 (3)	0.3967 (3)	0.0906 (11)	
H30A	0.3334	1.1276	0.3680	0.136*	
H30B	0.4237	1.0477	0.3529	0.136*	
H30C	0.4399	1.1355	0.4379	0.136*	
C37	0.0877 (2)	0.2872 (3)	0.22333 (18)	0.0644 (7)	
H37A	0.1336	0.3180	0.1950	0.077*	
C33	0.0311 (2)	0.2706 (3)	0.35331 (18)	0.0583 (7)	
H33A	0.0391	0.2901	0.4128	0.070*	
C29	0.4569 (2)	0.8816 (4)	0.4933 (3)	0.0996 (12)	
H29A	0.4352	0.8091	0.5225	0.149*	
H29B	0.5039	0.9346	0.5366	0.149*	
H29C	0.4888	0.8484	0.4510	0.149*	
O15	0.27212 (17)	0.4276 (3)	0.31983 (16)	0.0931 (7)	
C38	−0.1524 (2)	0.0859 (3)	0.1661 (2)	0.0852 (10)	
H38A	−0.1498	0.0752	0.1061	0.128*	
H38B	−0.2142	0.1288	0.1657	0.128*	
H38C	−0.1493	0.0010	0.1938	0.128*	
O1	0.31903 (13)	−0.28041 (18)	0.03724 (11)	0.0568 (5)	
C5	0.3453 (2)	−0.3965 (3)	0.09017 (19)	0.0605 (7)	
H5A	0.3779	−0.4587	0.0607	0.073*	
H5B	0.2847	−0.4376	0.0968	0.073*	

### Atomic displacement parameters (Å^2^)

**Table:**

	*U*^11^	*U*^22^	*U*^33^	*U*^12^	*U*^13^	*U*^23^
S1	0.0471 (3)	0.0582 (4)	0.0548 (4)	−0.0019 (3)	0.0085 (3)	0.0117 (3)
O3	0.0432 (9)	0.0706 (11)	0.0399 (9)	0.0006 (8)	0.0103 (7)	0.0064 (8)
O2	0.0449 (9)	0.0692 (11)	0.0350 (8)	−0.0111 (8)	0.0090 (7)	0.0003 (8)
O6	0.0482 (10)	0.0558 (10)	0.0632 (11)	−0.0051 (8)	0.0230 (9)	0.0019 (9)
O5	0.0488 (10)	0.0854 (13)	0.0957 (14)	0.0052 (11)	0.0212 (10)	−0.0239 (13)
C1	0.0411 (13)	0.0530 (13)	0.0404 (13)	−0.0054 (11)	0.0116 (11)	−0.0050 (11)
O4	0.0443 (10)	0.0787 (12)	0.0768 (13)	−0.0029 (10)	0.0092 (9)	−0.0219 (11)
C2	0.0422 (13)	0.0572 (14)	0.0406 (13)	−0.0010 (11)	0.0110 (11)	−0.0050 (11)
C13	0.0473 (13)	0.0458 (12)	0.0473 (14)	−0.0002 (11)	0.0134 (11)	0.0086 (11)
C12	0.0537 (15)	0.0609 (16)	0.0583 (16)	−0.0078 (13)	0.0250 (13)	−0.0027 (13)
C14	0.0586 (16)	0.0715 (17)	0.0536 (15)	−0.0091 (14)	0.0213 (13)	−0.0044 (13)
C3	0.0441 (14)	0.0722 (17)	0.0483 (14)	0.0015 (13)	0.0110 (12)	−0.0030 (13)
C8	0.0523 (15)	0.0699 (17)	0.0530 (16)	0.0053 (13)	0.0119 (13)	0.0028 (13)
O8	0.0530 (11)	0.0685 (12)	0.0960 (15)	0.0122 (10)	0.0171 (11)	0.0076 (11)
O7	0.0712 (12)	0.0898 (14)	0.0535 (11)	−0.0101 (12)	0.0065 (9)	0.0123 (11)
C6	0.0455 (13)	0.0599 (14)	0.0380 (13)	−0.0044 (11)	0.0101 (11)	0.0040 (11)
C4	0.0549 (16)	0.0600 (16)	0.0679 (19)	0.0063 (13)	0.0130 (14)	0.0035 (14)
C18	0.0576 (16)	0.0723 (18)	0.0554 (16)	0.0026 (14)	0.0227 (14)	0.0001 (14)
C16	0.0549 (17)	0.0755 (19)	0.076 (2)	−0.0036 (15)	0.0064 (15)	−0.0147 (17)
C17	0.0691 (19)	0.083 (2)	0.0536 (17)	0.0049 (17)	0.0095 (15)	−0.0107 (16)
C11	0.067 (2)	0.118 (3)	0.112 (3)	−0.018 (2)	0.030 (2)	−0.021 (2)
C7	0.0628 (17)	0.0686 (16)	0.0664 (18)	−0.0136 (14)	0.0186 (15)	0.0019 (15)
C15	0.0513 (16)	0.089 (2)	0.082 (2)	−0.0105 (15)	0.0273 (16)	−0.0107 (18)
C9	0.0438 (15)	0.088 (2)	0.071 (2)	0.0067 (15)	0.0112 (14)	−0.0128 (17)
C10	0.0609 (19)	0.164 (4)	0.086 (3)	0.014 (2)	0.0016 (18)	0.005 (3)
C19	0.060 (2)	0.160 (4)	0.139 (4)	−0.012 (3)	−0.004 (2)	−0.052 (3)
S2	0.0620 (4)	0.0451 (3)	0.0663 (4)	0.0042 (3)	0.0219 (3)	−0.0012 (3)
O11	0.0450 (9)	0.0576 (10)	0.0464 (10)	0.0058 (8)	0.0108 (8)	0.0128 (8)
O10	0.0472 (9)	0.0647 (11)	0.0468 (9)	−0.0001 (8)	0.0168 (8)	0.0141 (8)
O13	0.0489 (10)	0.0476 (9)	0.0644 (11)	−0.0047 (8)	0.0197 (9)	−0.0083 (8)
C22	0.0530 (15)	0.0384 (13)	0.0446 (14)	0.0023 (11)	0.0144 (12)	0.0041 (10)
O9	0.0688 (11)	0.0470 (9)	0.0364 (9)	0.0030 (8)	0.0099 (8)	−0.0003 (7)
O14	0.0850 (14)	0.0445 (10)	0.0727 (13)	0.0137 (10)	−0.0032 (11)	−0.0102 (9)
O16	0.0684 (9)	0.0575 (8)	0.0679 (9)	0.0060 (7)	0.0124 (7)	0.0032 (7)
C32	0.0615 (16)	0.0379 (12)	0.0535 (16)	0.0074 (11)	0.0168 (13)	−0.0010 (11)
C24	0.0666 (17)	0.0492 (14)	0.0492 (15)	0.0100 (13)	0.0173 (13)	−0.0109 (12)
O12	0.0608 (12)	0.0828 (13)	0.0628 (13)	−0.0097 (11)	0.0076 (10)	−0.0204 (11)
C28	0.0566 (17)	0.0554 (15)	0.0662 (18)	−0.0076 (13)	0.0143 (15)	−0.0122 (14)
C31	0.0577 (15)	0.0418 (12)	0.0502 (15)	0.0016 (11)	0.0110 (13)	0.0013 (11)
C25	0.0476 (14)	0.0702 (17)	0.0444 (14)	−0.0033 (13)	0.0134 (12)	0.0087 (13)
C21	0.0453 (13)	0.0428 (12)	0.0353 (13)	0.0031 (10)	0.0138 (11)	0.0004 (10)
C20	0.0506 (13)	0.0408 (11)	0.0386 (13)	0.0001 (10)	0.0154 (11)	0.0017 (10)
C34	0.0614 (16)	0.0693 (17)	0.0581 (17)	−0.0007 (15)	0.0191 (14)	0.0040 (15)
C23	0.0682 (18)	0.0345 (12)	0.0623 (18)	0.0064 (12)	0.0182 (15)	−0.0082 (12)
C35	0.0672 (17)	0.0543 (14)	0.0536 (17)	0.0073 (14)	0.0038 (14)	0.0042 (14)
C27	0.0708 (18)	0.095 (2)	0.0569 (17)	−0.0270 (18)	0.0178 (14)	−0.0003 (17)
C36	0.090 (2)	0.0724 (19)	0.0467 (16)	0.0062 (17)	0.0179 (15)	−0.0027 (14)
C26	0.0535 (17)	0.115 (3)	0.0705 (19)	0.0194 (17)	0.0243 (15)	0.0182 (18)
C30	0.093 (3)	0.069 (2)	0.115 (3)	−0.0268 (19)	0.040 (2)	−0.009 (2)
C37	0.083 (2)	0.0616 (16)	0.0538 (17)	0.0050 (16)	0.0291 (16)	0.0006 (14)
C33	0.0658 (17)	0.0652 (17)	0.0467 (14)	0.0042 (14)	0.0208 (14)	−0.0026 (13)
C29	0.060 (2)	0.118 (3)	0.109 (3)	0.014 (2)	0.0061 (19)	0.005 (2)
O15	0.0874 (16)	0.1010 (17)	0.1040 (17)	−0.0210 (14)	0.0486 (14)	−0.0096 (14)
C38	0.076 (2)	0.081 (2)	0.081 (2)	−0.0033 (18)	−0.0042 (18)	−0.0040 (18)
O1	0.0623 (11)	0.0611 (11)	0.0511 (10)	0.0026 (9)	0.0232 (9)	−0.0106 (9)
C5	0.0624 (18)	0.0503 (15)	0.0724 (19)	0.0027 (13)	0.0254 (16)	−0.0076 (14)

### Geometric parameters (Å, °)

**Table:**

S1—O7	1.416 (2)	S2—C32	1.749 (3)
S1—O8	1.424 (2)	O11—C21	1.424 (3)
S1—O6	1.5786 (19)	O11—C25	1.429 (3)
S1—C13	1.754 (3)	O10—C20	1.421 (3)
O3—C6	1.419 (3)	O10—C25	1.443 (3)
O3—C2	1.424 (3)	O13—C28	1.422 (3)
O2—C1	1.406 (3)	O13—C22	1.423 (3)
O2—C6	1.446 (3)	C22—C21	1.509 (3)
O6—C12	1.449 (3)	C22—C23	1.537 (4)
O5—C9	1.413 (4)	C22—H22A	0.9800
O5—C4	1.432 (3)	O9—C20	1.400 (3)
C1—O1	1.409 (3)	O9—C24	1.431 (3)
C1—C12	1.516 (4)	O14—C31	1.444 (3)
C1—C2	1.536 (3)	C32—C37	1.381 (4)
O4—C3	1.420 (3)	C32—C33	1.388 (3)
O4—C9	1.428 (3)	C24—C23	1.499 (4)
C2—C3	1.508 (3)	C24—H24A	0.9700
C2—H2A	0.9800	C24—H24B	0.9700
C13—C14	1.373 (3)	O12—C23	1.420 (3)
C13—C18	1.382 (4)	O12—C28	1.416 (3)
C12—H12A	0.9700	C28—C30	1.502 (4)
C12—H12B	0.9700	C28—C29	1.508 (4)
C14—C15	1.381 (4)	C31—C20	1.505 (3)
C14—H14A	0.9300	C31—H31A	0.9700
C3—C4	1.532 (4)	C31—H31B	0.9700
C3—H3A	0.9800	C25—C27	1.503 (4)
C8—C6	1.502 (4)	C25—C26	1.510 (4)
C8—H8A	0.9600	C21—C20	1.531 (3)
C8—H8B	0.9600	C21—H21A	0.9800
C8—H8C	0.9600	C34—C33	1.363 (4)
C6—C7	1.503 (4)	C34—C35	1.373 (4)
C4—C5	1.490 (4)	C34—H34A	0.9300
C4—H4A	0.9800	C23—H23A	0.9800
C18—C17	1.364 (4)	C35—C36	1.384 (4)
C18—H18A	0.9300	C35—C38	1.514 (4)
C16—C15	1.365 (4)	C27—H27A	0.9600
C16—C17	1.384 (4)	C27—H27B	0.9600
C16—C19	1.509 (4)	C27—H27C	0.9600
C17—H17A	0.9300	C36—C37	1.370 (4)
C11—C9	1.502 (5)	C36—H36A	0.9300
C11—H11A	0.9600	C26—H26A	0.9600
C11—H11B	0.9600	C26—H26B	0.9600
C11—H11C	0.9600	C26—H26C	0.9600
C7—H7B	0.9600	C30—H30A	0.9600
C7—H7C	0.9600	C30—H30B	0.9600
C7—H7D	0.9600	C30—H30C	0.9600
C15—H15A	0.9300	C37—H37A	0.9300
C9—C10	1.519 (5)	C33—H33A	0.9300
C10—H10A	0.9600	C29—H29A	0.9600
C10—H10B	0.9600	C29—H29B	0.9600
C10—H10C	0.9600	C29—H29C	0.9600
C19—H19A	0.9600	C38—H38A	0.9600
C19—H19B	0.9600	C38—H38B	0.9600
C19—H19C	0.9600	C38—H38C	0.9600
S2—O16	1.418 (2)	O1—C5	1.428 (3)
S2—O15	1.420 (2)	C5—H5A	0.9700
S2—O14	1.568 (2)	C5—H5B	0.9700
			
O7—S1—O8	120.45 (13)	C20—O10—C25	110.05 (16)
O7—S1—O6	108.85 (12)	C28—O13—C22	106.98 (18)
O8—S1—O6	103.72 (11)	O13—C22—C21	106.82 (17)
O7—S1—C13	108.79 (12)	O13—C22—C23	104.3 (2)
O8—S1—C13	109.66 (12)	C21—C22—C23	114.8 (2)
O6—S1—C13	104.10 (10)	O13—C22—H22A	110.2
C6—O3—C2	107.86 (17)	C21—C22—H22A	110.2
C1—O2—C6	110.70 (17)	C23—C22—H22A	110.2
C12—O6—S1	116.95 (14)	C20—O9—C24	114.55 (18)
C9—O5—C4	107.4 (2)	C31—O14—S2	118.85 (17)
O2—C1—O1	110.23 (19)	C37—C32—C33	120.0 (3)
O2—C1—C12	110.8 (2)	C37—C32—S2	120.1 (2)
O1—C1—C12	101.59 (17)	C33—C32—S2	119.9 (2)
O2—C1—C2	104.05 (17)	O9—C24—C23	110.54 (19)
O1—C1—C2	114.3 (2)	O9—C24—H24A	109.5
C12—C1—C2	116.0 (2)	C23—C24—H24A	109.5
C3—O4—C9	107.3 (2)	O9—C24—H24B	109.5
O3—C2—C3	108.1 (2)	C23—C24—H24B	109.5
O3—C2—C1	103.14 (18)	H24A—C24—H24B	108.1
C3—C2—C1	115.84 (19)	C23—O12—C28	109.0 (2)
O3—C2—H2A	109.8	O12—C28—O13	104.4 (2)
C3—C2—H2A	109.8	O12—C28—C30	111.6 (2)
C1—C2—H2A	109.8	O13—C28—C30	110.3 (3)
C14—C13—C18	120.4 (2)	O12—C28—C29	108.3 (3)
C14—C13—S1	121.2 (2)	O13—C28—C29	108.4 (2)
C18—C13—S1	118.40 (19)	C30—C28—C29	113.4 (3)
O6—C12—C1	109.05 (18)	O14—C31—C20	106.99 (19)
O6—C12—H12A	109.9	O14—C31—H31A	110.3
C1—C12—H12A	109.9	C20—C31—H31A	110.3
O6—C12—H12B	109.9	O14—C31—H31B	110.3
C1—C12—H12B	109.9	C20—C31—H31B	110.3
H12A—C12—H12B	108.3	H31A—C31—H31B	108.6
C13—C14—C15	118.9 (3)	O11—C25—O10	104.82 (18)
C13—C14—H14A	120.5	O11—C25—C27	112.1 (2)
C15—C14—H14A	120.5	O10—C25—C27	109.8 (2)
O4—C3—C2	107.9 (2)	O11—C25—C26	107.5 (2)
O4—C3—C4	104.43 (19)	O10—C25—C26	108.8 (2)
C2—C3—C4	114.7 (2)	C27—C25—C26	113.4 (2)
O4—C3—H3A	109.9	O11—C21—C22	108.62 (18)
C2—C3—H3A	109.9	O11—C21—C20	103.90 (17)
C4—C3—H3A	109.9	C22—C21—C20	114.7 (2)
C6—C8—H8A	109.5	O11—C21—H21A	109.8
C6—C8—H8B	109.5	C22—C21—H21A	109.8
H8A—C8—H8B	109.5	C20—C21—H21A	109.8
C6—C8—H8C	109.5	O9—C20—O10	109.87 (17)
H8A—C8—H8C	109.5	O9—C20—C31	102.71 (18)
H8B—C8—H8C	109.5	O10—C20—C31	111.34 (19)
O3—C6—O2	104.48 (17)	O9—C20—C21	115.11 (19)
O3—C6—C8	111.6 (2)	O10—C20—C21	103.74 (18)
O2—C6—C8	108.8 (2)	C31—C20—C21	114.26 (18)
O3—C6—C7	108.6 (2)	C33—C34—C35	121.9 (3)
O2—C6—C7	110.0 (2)	C33—C34—H34A	119.0
C8—C6—C7	113.1 (2)	C35—C34—H34A	119.0
O5—C4—C5	108.3 (2)	O12—C23—C24	109.5 (2)
O5—C4—C3	104.5 (2)	O12—C23—C22	104.2 (2)
C5—C4—C3	112.8 (2)	C24—C23—C22	112.4 (2)
O5—C4—H4A	110.3	O12—C23—H23A	110.2
C5—C4—H4A	110.3	C24—C23—H23A	110.2
C3—C4—H4A	110.3	C22—C23—H23A	110.2
C17—C18—C13	119.3 (2)	C34—C35—C36	117.9 (3)
C17—C18—H18A	120.3	C34—C35—C38	121.5 (3)
C13—C18—H18A	120.3	C36—C35—C38	120.6 (3)
C15—C16—C17	118.2 (3)	C25—C27—H27A	109.5
C15—C16—C19	121.0 (3)	C25—C27—H27B	109.5
C17—C16—C19	120.8 (3)	H27A—C27—H27B	109.5
C18—C17—C16	121.4 (3)	C25—C27—H27C	109.5
C18—C17—H17A	119.3	H27A—C27—H27C	109.5
C16—C17—H17A	119.3	H27B—C27—H27C	109.5
C9—C11—H11A	109.5	C37—C36—C35	121.6 (3)
C9—C11—H11B	109.5	C37—C36—H36A	119.2
H11A—C11—H11B	109.5	C35—C36—H36A	119.2
C9—C11—H11C	109.5	C25—C26—H26A	109.5
H11A—C11—H11C	109.5	C25—C26—H26B	109.5
H11B—C11—H11C	109.5	H26A—C26—H26B	109.5
C6—C7—H7B	109.5	C25—C26—H26C	109.5
C6—C7—H7C	109.5	H26A—C26—H26C	109.5
H7B—C7—H7C	109.5	H26B—C26—H26C	109.5
C6—C7—H7D	109.5	C28—C30—H30A	109.5
H7B—C7—H7D	109.5	C28—C30—H30B	109.5
H7C—C7—H7D	109.5	H30A—C30—H30B	109.5
C16—C15—C14	121.7 (3)	C28—C30—H30C	109.5
C16—C15—H15A	119.1	H30A—C30—H30C	109.5
C14—C15—H15A	119.1	H30B—C30—H30C	109.5
O5—C9—O4	104.4 (2)	C36—C37—C32	119.2 (3)
O5—C9—C11	108.8 (3)	C36—C37—H37A	120.4
O4—C9—C11	108.5 (3)	C32—C37—H37A	120.4
O5—C9—C10	110.9 (3)	C34—C33—C32	119.3 (2)
O4—C9—C10	110.8 (3)	C34—C33—H33A	120.3
C11—C9—C10	113.2 (3)	C32—C33—H33A	120.3
C9—C10—H10A	109.5	C28—C29—H29A	109.5
C9—C10—H10B	109.5	C28—C29—H29B	109.5
H10A—C10—H10B	109.5	H29A—C29—H29B	109.5
C9—C10—H10C	109.5	C28—C29—H29C	109.5
H10A—C10—H10C	109.5	H29A—C29—H29C	109.5
H10B—C10—H10C	109.5	H29B—C29—H29C	109.5
C16—C19—H19A	109.5	C35—C38—H38A	109.5
C16—C19—H19B	109.5	C35—C38—H38B	109.5
H19A—C19—H19B	109.5	H38A—C38—H38B	109.5
C16—C19—H19C	109.5	C35—C38—H38C	109.5
H19A—C19—H19C	109.5	H38A—C38—H38C	109.5
H19B—C19—H19C	109.5	H38B—C38—H38C	109.5
O16—S2—O15	118.05 (14)	C1—O1—C5	114.93 (18)
O16—S2—O14	108.29 (12)	O1—C5—C4	110.5 (2)
O15—S2—O14	109.45 (15)	O1—C5—H5A	109.5
O16—S2—C32	110.89 (12)	C4—C5—H5A	109.5
O15—S2—C32	110.16 (14)	O1—C5—H5B	109.5
O14—S2—C32	98.14 (11)	C4—C5—H5B	109.5
C21—O11—C25	106.47 (17)	H5A—C5—H5B	108.1

### Hydrogen-bond geometry (Å, °)

**Table:**

*D*—H···*A*	*D*—H	H···*A*	*D*···*A*	*D*—H···*A*
C3—H3A···O13^i^	0.98	2.70	3.358 (3)	125
C4—H4A···C17^i^	0.98	2.83	3.657 (5)	142
C21—H21A···O3^ii^	0.98	2.58	3.456 (3)	149
C23—H23A···O16^ii^	0.98	2.62	3.466 (3)	145
C23—H23A···C33^ii^	0.98	2.89	3.738 (4)	145
C37—H37A···O8	0.93	2.61	3.341 (4)	136

Symmetry codes: (i) *x*, *y*−1, *z*; (ii) *x*, *y*+1, *z*.

## References

[bb1] Bruker (2001). *SAINT-Plus* Bruker AXS Inc., Madison, Wisconsin, USA.

[bb2] Bruker (2005). *APEX2* Bruker AXS Inc., Madison, Wisconsin, USA.

[bb3] Dekany, G., Lundt, I., Niedermair, F., Bichler, S., Spreitz, J., Sprenger, F. K. & Stutz, A. E. (2007). *Carbohydr. Res.* **342**, 1249–1253. 10.1016/j.carres.2007.02.026 17368437

[bb4] Flack, H. D. (1983). *Acta Cryst.* A**39**, 876–881.

[bb5] Hirst, E. L., Mitchelle, W. E. A., Percival, E. E. & Percival, E. G. V. (1953). *J. Chem. Soc.* pp. 3170–3175.

[bb6] Lis, T. & Weichsel, A. (1987). *Acta Cryst.* C**43**, 1954–1956.

[bb7] Reitz, A. R., Tuman, R. W., Marchione, C. S., Jordan, A. D. Jr, Bowden, C. R. & Maryanoff, B. E. (1989). *J. Med. Chem.* **32**, 2110–2116. 10.1021/jm00129a015 2769683

[bb8] Sheldrick, G. M. (2008). *Acta Cryst.* A**64**, 112–122. 10.1107/S0108767307043930 18156677

